# Amitraz Poisoning: The not so (Un)common Poisoning

**DOI:** 10.7759/cureus.5438

**Published:** 2019-08-20

**Authors:** Manish Bhartiya, Bandhul Hans, Sambit Sundaray, Amitabh Sagar

**Affiliations:** 1 Neurology, All India Institute of Medical Sciences, New Delhi, IND; 2 Forensic Medicine, All India Institute of Medical Sciences, New Delhi, IND; 3 Internal Medicine, Military Hospital Ahmednagar, Ahmednagar, IND; 4 Internal Medicine, Command Hospital, Chandimandir, New Delhi, IND

**Keywords:** op poisoning, amitraz poisoning, insecticide poisoning, organophosphate poisoning

## Abstract

Amitraz is a widely used insecticide and antiparasitic drug. It can cause poisoning in humans via oral, inhalation, and dermal routes. Clinical features hence produced may mimic organophosphate (OP) poisoning because of several shared features (miosis, bradycardia, hypotension) along with a history of possible insecticide poisoning. But the presence of hyperglycemia, hypothermia, and reduced gastrointestinal motility along with normal serum cholinesterase levels and the absence of fasciculations and a hypersecretory state (salivation, lacrimation, perspiration, and diarrhea) point against OP poisoning. Analysis of the poison container also helps confirm the poison. Management is mostly supportive with a good prognosis.

## Introduction

Amitraz finds a commonplace use in pharmaceutical, veterinary and agricultural industries as an acaricide, insecticide, and antiparasitic. The common brand name available for use in our country is "RIDD" though it is used worldwide under names of Avartin, Baam, Mitaban, Triatox, Triazid, Topline, Tudy, Ectodex, Danicut, Mitac, Amitraze. Chemically it is 1,5-di-(2,4-dimethylphenyl)-3-methyl-1,3,5-triaza-penta-1,4-diene, a member of the formamidine pesticide [1]. The US Environmental Protection Agency classifies amitraz as slightly toxic by the oral and inhalation routes (Toxicity Category III) and moderately toxic by the dermal route (Toxicity Category II) [2]. There have been very few case-reports from our country till date mostly due to underreporting of cases. This is further compounded by the fact that the toxidrome mimics organophosphate (OP) poisoning and is inappropriately treated with an uneventful recovery in most cases [3-5]. Till date, only six deaths have been reported in the literature due to amitraz poisoning [6]. We report two cases of amitraz poisoning managed at our center with variable outcomes at the end.

## Case presentation

Case 1

A 61-year-old male was transferred to our center from a peripheral hospital as a case of OP poisoning for further management. He was a chronic alcohol consumer (> 160-180 mg/day) for the past 30 years and had accidentally consumed an unquantifiable amount of insecticide along with a binge of alcohol. He became drowsy and disoriented and was hence admitted to hospital. Initial clinical evaluation revealed the patient to be drowsy, hypotensive, and had tachycardia. On examination, he had miosis and tachycardia. In view of the clinical presentation, he was started on management for OP with gastric lavage, intravenous (IV) fluids, vasopressors, broad-spectrum antibiotics, atropine, pralidoxime (PAM), and blood component support. However, he responded poorly to atropine and PAM and continued to deteriorate.

During the hospital stay, the patient developed hemolysis and acute kidney injury (AKI), which was managed with hemodialysis. He also developed severe hemolytic anemia requiring transfusions, which has been described in the literature with xylene (solvent for amitraz) poisoning [7]. He also required mechanical ventilatory support due to poor sensorium. Meanwhile, a review of the insecticide revealed it to be amitraz. Also supporting against the diagnosis of OP poisoning were the normal serum choline-esterase levels - 8432 IU/L (normal range: 2710 to 11510 IU/L).

Electrocardiogram (ECG) showed normal sinus rhythm, and imaging studies showed cardiomegaly on chest radiograph and normal-sized kidneys with increased cortical echogenicity, perirenal collection and fatty liver on abdominal ultrasonography.

His baseline and subsequent laboratory investigations are listed in Table 1.

**Table 1:** Baseline and follow up investigations for case 1

	Baseline	Day 2	Day 3	Day 4	Day 5	Day 6
Hemoglobin (g/dl)	8.3	6.8	6.8	7.5	7.6	7.3
Total Leukocyte Count (cells/mm^3^)	16300	6100	13900	23100	19800	16600
Platelet (x10^3 ^cells/mm^3^)	102	118	107	59	72	61
Urea/Creatinine (mg/dl)	40/0.4	42/1.0	48/1.2	85/2.06	108/2.6	171/4.8
Serum LDH (U/L)	3046					
pH	7.29					
PaCO2 (mmHg)	72					
HCO3- (mmol/l)	17.30					

The patient was managed with central venous pressure (CVP) guided fluids, hematinics, antibiotics, and supportive measures. He made an uneventful recovery in one week. Most cases recover entirely with aggressive supportive treatment.

Case 2

A 62-year-old female, known case of primary hypertension, was brought in at a tertiary care center with a history of alleged consumption of a pesticide, details of which were not available. She came in at the tertiary center followed by immediate loss of consciousness. Her relatives had also noticed profuse diaphoresis, frothing at the mouth and high colored urine. No history of generalized tonic-clonic movements, loose stools, hematemesis, melena, hematuria, or oliguria was present. Initial evaluation revealed the patient to be hemodynamically stable with a respiratory rate of 26/min, oxygen saturation (SpO2) 86% on room air. Excessive oral secretions were noticed, and pupils were bilaterally constricted. Chest examination revealed bilateral diffuse crackles. She received saline gastric lavage, IV fluids, oxygen supplementation. She was atropinised in view of miosis and intermittent bradycardia with excessive oral and respiratory secretions. During the course of hospital stay, she developed labored breathing and desaturation for which she was intubated and placed on a mechanical ventilator. The management for OP poisoning was stopped after reviewing the composition of the insecticide, which revealed it to be amitraz.

She was then transferred to our center for further management. On examination at our center patient was afebrile with a heart rate of 90/min and blood pressure of 138/90 mm Hg. Grade II bedsores were present. Glasgow coma score (GCS) was nine. Right pupil showed normal reaction whereas the left remained constricted. Horizontal and vertical gaze palsy along with skew deviation in the left eye was present. There was spastic quadriparesis with bilaterally extensor plantar response, deep tendon reflexes (DTRs) were bilaterally exaggerated. Chest examination revealed bilateral normal air entry. Rest of the examination was within normal. Investigations done at our center revealed the figures (Table 2).

**Table 2:** Follow up Investigations for case 2

Hemoglobin (g/dl)	12.6
Total Leukocyte Count (cells/mm^3^)	19000
Platelet (x10^3^ cells/mm^3^)	220
Serum Sodium (mEq/L)	138
Serum Potassium (mEq/L)	4.0
Urea/Creatinine (mg/dl)	39/0.8
Serum total/direct bilirubin (mg/dl)	0.8/0.5
Serum albumin (g/dl)	3.0
Aspartate transaminase (U/L)	52
Alkaline phosphatase (U/L)	142

A peripheral blood smear done showed neutrophilic leukocytosis, and an MRI brain revealed Osmotic Demyelination Syndrome.

She was managed at our center with IV fluids, Ryles tube feed, IV antibiotics, and supportive measures. She was given supportive care; but because of the central nervous system (CNS) depression and respiratory failure, she was placed on ventilatory support. During hospitalization, she developed hypokalemia (2.7 mEq/L), which was corrected with IV and oral supplements. In view of expected prolonged mechanical ventilation, she was tracheostomised. On day 11 of admission, she developed sudden onset bradycardia and hypotension. Cardiopulmonary resuscitation (CPR) as per advanced cardiac life support (ACLS) protocol was initiated; however, despite all measures, she succumbed to her illness. Severe bradycardia responding promptly to atropine boluses have been described in previous case reports but the event, in this case, could have multifactorial etiology like dyselectrolytemia, respiratory failure, sick sinus or underlying coronary and hypertensive heart disease.

## Discussion

Amitraz is increasingly being used worldwide in veterinary medicine and agriculture. Poisoning may occur by oral ingestion, inhalation, and dermal routes. The present knowledge about formamidines is frequently built on animal studies because of the limited human intoxication studies. The toxicity from this poisoning can be attributed to both amitraz and the solvent, xylene [5,7-9]. Although the ingested dose of amitraz cannot be determined because it is diluted at one part in 500 before usage, the proposed lethal dose is 200 mg/kg [10]. Accordingly, with an average adult weight of 60 kg, a dose of 12 g is supposedly lethal.

Amitraz is an α2 adrenergic agonist and mimics clonidine in its manifestations. It acts as an agonist on both pre- and postsynaptic α2-adrenergic receptors [11,12]. Presynaptic receptor stimulation inhibits norepinephrine discharge, while stimulation of postsynaptic receptors leads to effects similar to α1-stimulation [11,13]. This is what produces the most of the clinical manifestations, altered sensorium (83%), miosis (50%), bradycardia (47%), vomiting (36%), respiratory failure (34%) and hypotension (31%) [6]. Hypothermia (24%) due to inhibition of Prostaglandin E2 synthesis and hyperglycemia (48%) are distinctive features of this poisoning [6]. Miosis may be seen due to the presynaptic effect at low doses, or rarely mydriasis may also be seen due to postsynaptic effect at higher doses.

Amitraz has been shown to increase plasma glucose levels and suppress insulin release through its effect on α2 receptors in animal studies [14]. Seizures have been reported by Yilmaz et al. and Ertekin et al. [8,10]. Amitraz is a potent hepatotoxic drug which acts by decreasing hepatic glutathione activity, but the liver enzymes have been shown to return to baseline within 48 hours [10]. It is a potent inhibitor of liver monoamine oxidase enzyme in rats. The solvent, xylene, may additionally cause acute toxic signs like CNS depression, ataxia, nystagmus, stupor, coma, and episodes of neuroirritability [5,7-9].

The most common clinical features associated with amitraz poisoning often lead clinicians to misdiagnose it as the much commoner OP poisoning given the history of suspected insecticide poisoning. However, there are important distinguishing clinical features that must be looked for in all cases of suspected OP poisoning. Presence of hyperglycemia, hypothermia, and reduced gastrointestinal motility with the absence of fasciculations and a hypersecretory state (salivation, lacrimation, perspiration, and diarrhea) point against OP poisoning [15,16]. Although less reliable, the presence of a solvent smell or mothball like smell points to amitraz poisoning versus a garlicky odor in OP poisoning [17]. Investigating red blood cell (RBC) or serum cholinesterase levels helps distinguish the two clearly (RBC cholinesterase levels are a better marker than serum cholinesterase levels), with normal cholinesterase levels in amitraz poisoning and low levels in OP poisoning [3, 18, 19].

As there is no specific antidote for amitraz poisoning, medical management is essentially symptomatic and supportive. The main approach while treating the patients of amitraz intoxication includes hemodynamic stabilization by proper hydration, maintaining airway, oxygen administration, reducing the absorption of poisonous material and measures to improve elimination of the toxin from the body [6,8]. Amitraz poisoning carries a good prognosis with a low case fatality rate (2%), despite the severe life-threatening clinical features [6]. Although associated with a good prognosis, the etiology of mortality in the second case could have been multifactorial, like dyselectrolytemia, respiratory failure, sick sinus or underlying coronary and hypertensive heart disease. There have been previous case reports describing severe bradycardia responding promptly to atropine boluses.

## Conclusions

Amitraz intoxication mimics OP poisoning in a lot of ways and hence may be misdiagnosed. It can be differentiated from OP poisoning by the presence of a ‘mothball-like’ odor, hyperglycemia, hypothermia and/or reduced gastrointestinal motility, absence of fasciculations and hypersecretory state (salivation, lacrimation, perspiration, and diarrhea). A normal serum cholinesterase level helps in ruling out OP poisoning. Investigating the poison container becomes of paramount importance in such intoxications for correct identiﬁcation. Good supportive management is the only treatment option for the lack of a specific antidote but is associated with favorable clinical outcomes. Although the reported fatality rate is 2%, the actual rate may be more due to failure to correctly diagnose the poisoning.
